# The Principal Forms of the Skeleton and of the Teeth

**Published:** 1854-10

**Authors:** 


*The principal Forms of the Skeleton and of the Teeth.* By Professor R. OWEN, F. R. S., &c. &c, Author of "Odontography," "Lectures on comparative Anatomy," "Archetype of the Skeleton," "On the nature of *Limbs*," "History of British Fossil Mammalia, &c &c." Philadelphia, Blanchard and Lea, 1854.

This is also an Octavo Volume of 229 pages, re-published from the first London edition. The author is one of the most distinguished cultivators of natural history and comparative anatomy; and his book though brief, is nevertheless highly interesting and valuable. Like the preceding, it was written chiefly for the general reader and they both constitute a part of a series of works published in London, and styled "Orr's Circle of the Sciences." The present volume will well repay a careful perusal even by the professional reader.

